# The Use of Acoustic Emission to Determine the Safe Range of Operational Stresses of 3D-Printed ABS Polymer Components

**DOI:** 10.3390/ma18214834

**Published:** 2025-10-22

**Authors:** Krzysztof Dudzik, Patryk Krawulski, Robert Starosta, Burkhard Ziegler

**Affiliations:** 1 Faculty of Marine Engineering, Gdynia Maritime University, 81-225 Gdynia, Poland; p.krawulski@wm.umg.edu.pl (P.K.); r.starosta@wm.umg.edu.pl (R.S.); 2 Mechanical Engineering and Energy Technology Department, Technische Hochshule Mittlehessen—University of Applied Sciences, 35390 Giessen, Germany; burkhard.ziegler@me.thm.de

**Keywords:** 3D printing, ABS polymer, strength tests, acoustic emission, destruction of materials, a safe load range

## Abstract

This work proposes using acoustic emission during a static tensile test to determine the stress characteristics of the initial phase of the destruction process of elements printed using the material extrusion (MEX) additive method at various printing parameters. The changed parameters were layer height, print orientation, filling ratio, and nozzle temperature. ABS material was chosen for printing. The experiment was carried out according to the Taguchi plan. The analysis of the results showed that changes in printing parameters significantly impact the mechanical properties of the tested elements. The parameter that had the greatest impact on strength was the filling ratio. Maximum tensile strength was achieved with the following printing parameters: 0.24 mm layer, 30°, 100% infill, 275 °C, concentric pattern. The results can be the basis for optimizing the additive printing process and improving the efficiency and reliability of manufactured components. The results of recorded acoustic emissions during strength tests allow the identification of stresses characteristic of the initial phase of the destruction process of the tested material. This phase is the elastic-visco-plastic transition, and the use of the AE method enables its detection 2–5 s earlier than the static tensile test. This allows us to determine the safe range of stresses when using the mentioned materials, which is particularly helpful in designing structures or spare parts. The test results showed that the critical stress for the investigated components is approximately 6 MPa, and exceeding this value is associated with the risk of unsafe operation.

## 1. Introduction

**Figure 1:**
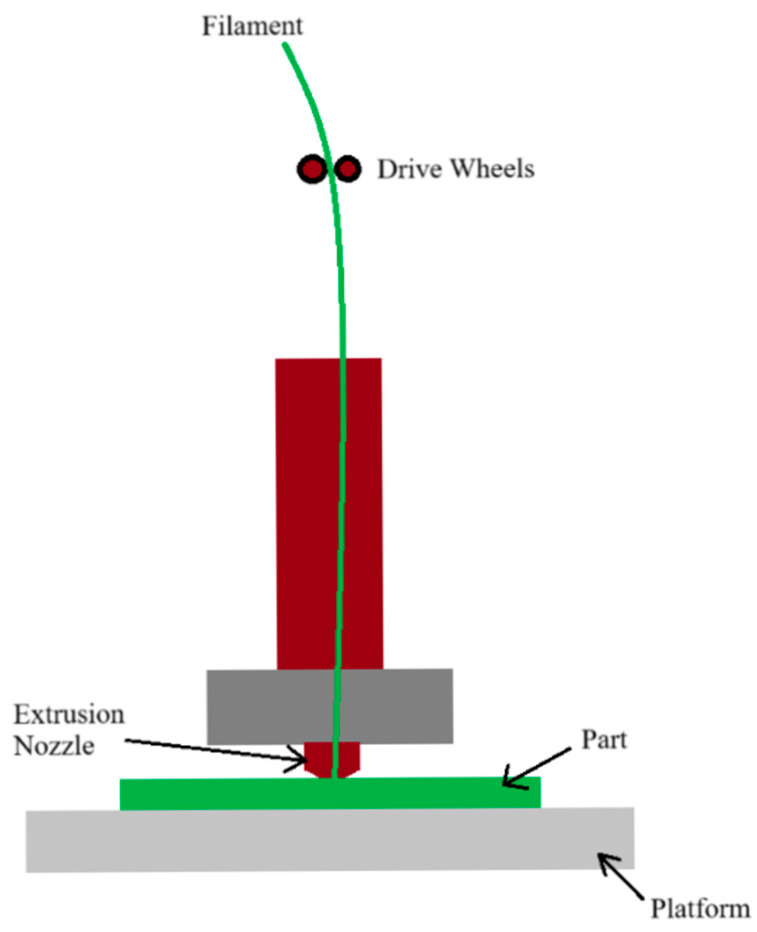
The idea of material extrusion (MEX) system drawing.

3D printing, especially the additive material extrusion (MEX) method according to ISO 52900 [1], has become one of the key tools in modern production. It enables the quick and economical production of complex components [2]. Additive technology can be divided into different subfields: stereolithography (SLA), fused fiber fabrication (FFF), laser sintering (SLS), electron beam melting, laser melting and hybrid processes [3]. Additive shaping involves the layer-by-layer application of material (Figure 1), which leads to the creation of the desired 3D object [4]. In material extrusion printers, the head moves on the X and Y axes, and the up and down movement is performed by the platform (Z axis). The filament is heated, melted and then extruded through a nozzle [5]. The first step when using the additive material extrusion (MEX) technique is to design a 3D model using special CAD software. After the design is completed, the model is converted to an STL file [6]. The result of creating an STL file is the ability to generate a G-code file with which the operator can start the printing process [7].

Maintenance cost in material extrusion (MEX) production is one of the key advantages compared to other technologies [8]. The material often used in this technology is ABS (acrylonitrile-butadiene-styrene). This material has high strength properties, impact resistance, and resistance to various types of chemicals [9]. These features make it an ideal choice for components that require durability and precision, such as marine parts, automotive parts, electronics housings, and tool components [10]. It is therefore necessary to predict how parts will behave under the influence of loads [11]. Despite many advantages, one of the challenges remains to ensure the reliability and safety of components made of ABS in various operating conditions [12].

Numerous studies have analyzed how selected material extrusion (MEX) printing parameters affect the mechanical and structural performance of ABS parts. For example, research has shown that layer height significantly influences tensile strength and surface quality, with thinner layers generally improving strength due to better interlayer adhesion [13]. Printing orientation also plays a crucial role—samples printed along the Z-axis tend to show lower tensile strength compared to those printed in the XY plane, due to weaker bonding between layers [14]. The infill density directly affects stiffness and impact resistance, with higher infill ratios increasing overall mechanical performance but also extending printing time and material use. Similarly, nozzle temperature influences the degree of polymer fusion, where insufficient temperature leads to poor bonding and internal defects, while excessive heat may degrade material properties [15]. The surface finishing and cooling conditions also impact fatigue behavior and dimensional accuracy. These findings highlight the complexity of parameter optimization in material extrusion (MEX) and justify the need for further analysis to determine safe and efficient process settings for ABS components.

The acoustic emission (AE) method has gained importance as a tool for monitoring the technical condition of materials and detecting microcracks and other structural defects [16]. AE involves recording sound waves emitted by the material when damage occurs [17]. With the popularization of the application of materials in various fields, there is a need to monitor changes in their structures during operation [18]. This method is classified as a non-destructive method and can be used to diagnose the destruction of materials. Diagnosis can be carried out in laboratory and operational conditions [19]. AE is a phenomenon in which elastic waves are generated, forced by the impact resulting from structural changes (e.g., fiber cracking in laminates) [16,17], metals and their bonded joints [20], in concrete [21], and rocks (e.g., cracking under variable temperature conditions) [22]. Acoustic emission waves are detected by piezoelectric sensors on the surface of the tested structure, which have the function of changing the strain energy into an electrical signal [23].

Determining the safe load range of the structure is crucial for ensuring reliable operation and safe operation. For this purpose, a safety factor is determined, which usually refers to the strength properties determined in a static tensile test. This procedure has certain limitations in the case of anisotropic materials, which include elements manufactured by the MEX method. Determining the first symptoms of the destruction process in laboratory conditions allows for the design of appropriate operating conditions for the tested structure. Such a designated range of safe use seems to be more appropriate than the classic determination of the safety factor concerning such parameters as, most often, tensile strength or, in more precise tests, the transition stress to the visco-plastic state.

Acoustic emission (AE) measurement is widely used to monitor damage initiation and propagation in fiber-reinforced polymer composites due to its sensitivity to phenomena such as matrix microcracks, delamination, and fiber breakage. Signal descriptors enable the differentiation of damage mechanisms and the identification of stress thresholds associated with material degradation. Compared to other non-destructive techniques (e.g., active thermography [24], Raman spectroscopy [25], digital image correlation [26], and metric entropy analysis [27]) used to determine critical stress levels in materials under load, AE has proven to be more sensitive and capable of identifying such changes at earlier stages.

For material extrusion (MEX)-manufactured polymer components, there is a significant lack of published results on the simultaneous acquisition of AE signals and the determination of their mechanical properties. The authors identified only one publication by García-Vilana et al. [28], in which AE activity was correlated with stiffness loss and microstructural damage development in PLA polymer samples during mechanical testing. The study demonstrated the effectiveness of AE in detecting delamination, monitoring crack propagation under tensile loading, and optimizing printing parameters.

The article aims to investigate the possibility of using acoustic emission to determine the safe range of use for elements made from ABS material with the material extrusion (MEX) method. A review of the available literature and the preliminary experimental studies, along with their statistical analysis, revealed that all the examined parameters had a significant influence on the mechanical properties of the printed components. The research analysis focuses on the impact of selected printing parameters that statistically had the greatest impact on the properties of the final prints. The selected parameters include: print layer height, sample orientation (X-axis), filling ratio, nozzle temperature, and type of surface layer finish. The research results are intended to develop recommendations for optimizing the printing process, which may contribute to increasing the reliability and safety of manufactured components.

## 2. Materials and Methods

**Figure 2:**
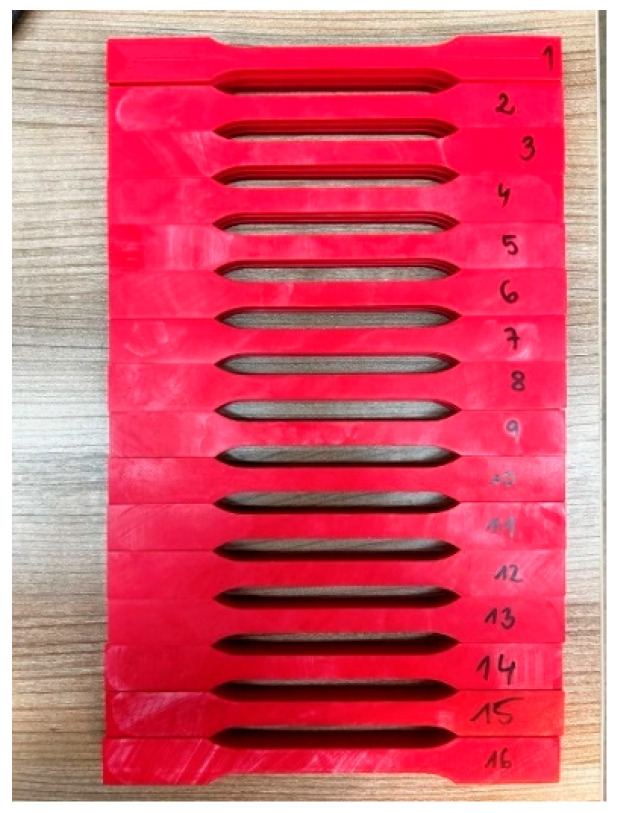
Photo of samples for static tensile testing.

**Figure 3:**
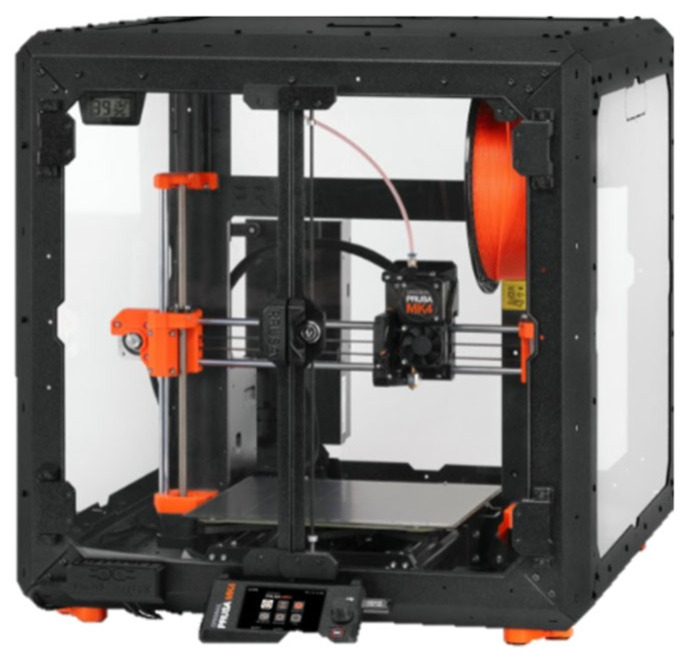
View of the Original Prusa iMK3S+ printer.

**Figure 4:**
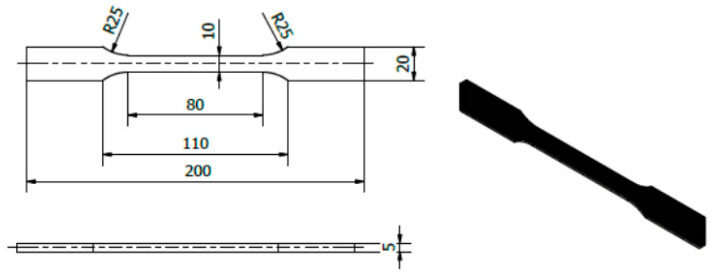
View and dimensions of the sample for strength tests.

An ABS filament with a diameter of 1.75 mm was used to produce the samples. The samples (Figure 2) were based on changes in selected printing parameters, which were made using an Original Prusa iMK3S+ 3D (Prusa Research a.s., Prague, Czech Republic) printer (Figure 3). Samples for the static tensile test (Figure 4) were made in accordance with the PN-ISO 5893:2015-12 standard [29] based on the Taguchi orthogonal plan. Strength tests were carried out on a Zwick & Roell 100 kN testing machine (ZwickRoell GmbH & Co KG, Ulm, Germany). Each trial was repeated three times to limit the randomness of the results.

**Figure 5:**
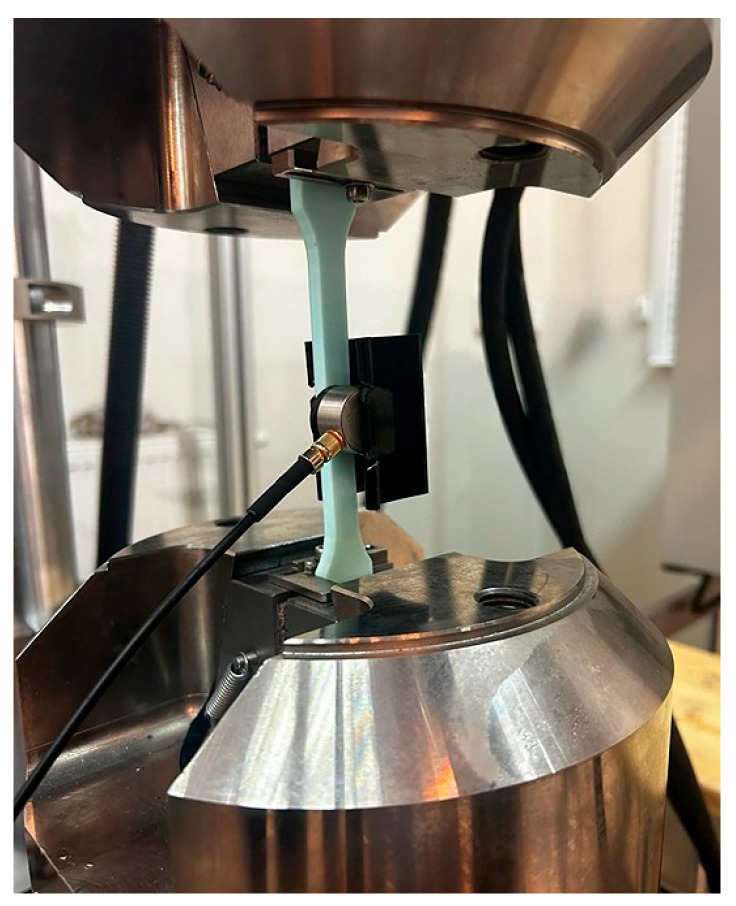
View of the sample during a static tensile test with the AE sensor.

**Figure 6:**
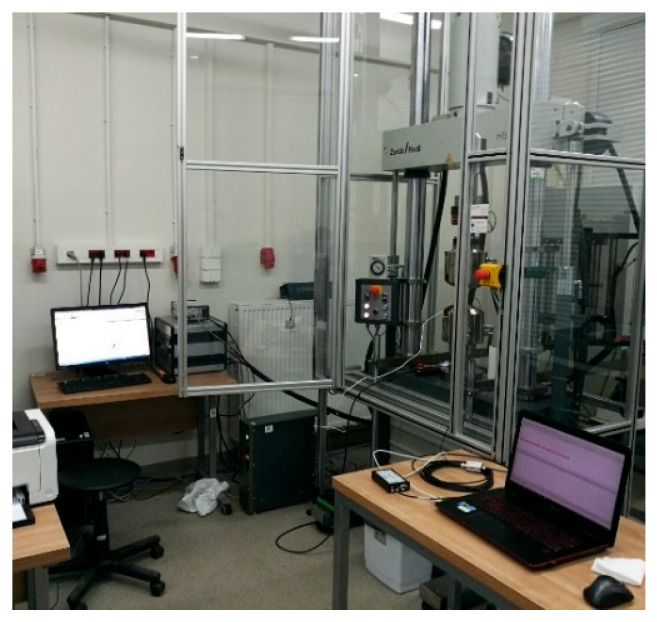
View of the test stand for carrying out strength and AE tests.

AE tests were carried out using a set consisting of: a single-channel USB AE Node recorder (Physical Acoustics Corporation, Princeton, NJ, USA), type 1283 with a bandwidth of 20 kHz–1 MHz; a preamplifier with a bandwidth of 75 kHz–1.1 MHz; an AE-Sensor VS150M (Vallen Systeme, Icking, Germany) (in the frequency range of 100–450 kHz) (Figure 5); and a computer with AE Win with USB Version E5.30 (Figure 6). Silicone grease was used as a coupling between the specimen surface and the AE sensor. The sensor was mounted on the samples using a spring holder to ensure a constant pressure force on the test surface. Software (AE Win with USB Version E5.30) dedicated to the selected set for recording and analyzing AE data was used. The tests were carried out in accordance with the acoustic emission testing standard (PN-EN 1330-9:2017-09 [30]; PN-EN 13554:2011E [31]).

Selected printing parameters are presented in Table 1, while Table 2 shows the Taguchi plan of experiment.

## 3. Results and Discussion

### 3.1. Statistical Analysis

Using analysis of variance, it was demonstrated that all independent variables have a statistically significant impact on tensile strength, as indicated by the analysis of variance test (F and *p* values in Table 3). The total sum of squares is 190.511, of which only 0.1468 of the sums of squares are not explained by the input parameters, which is 0.00077%. The sum of squares analysis shows that the filling ratio has the greatest influence on tensile strength, while the nozzle temperature has the least influence. Table 3 presents the analysis of variance of the input parameters.

The indicated values of the significance coefficient (Table 3), less than 0.01, prove that the printing parameters were correctly adopted.

Taguchi analysis presents two types of factors: noise factors and control factors. Noise factors are those that are difficult or impossible to control, while control factors are determined by the researcher. Therefore, in the Taguchi method, the signal-to-noise (S/N) ratio is used to create a process that becomes less sensitive to changes in noise factors. The goal is to achieve the maximum strength of the tested material. In a static tensile test, the tensile strength must have a high value, therefore the “larger is better” ratio (S/N) is calculated from Equation (1).

(1) $$S/N=-10\;\log\;[\frac{1}{n}\sum_{i=1}^{n}\frac{1}{y_{i}^{2}}]$$

Table 4 shows the average results of the ultimate tensile strength (UTS) in the static tensile test of samples made of ABS material and the values of the S/N ratio.

**Figure 7:**
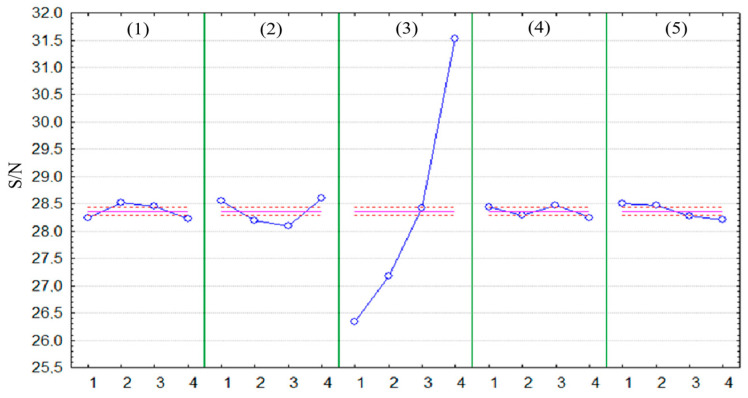
Average values of the S/N ratio for individual printing parameters; blue lines—the influence of the selected parameter on the S/N value; pink lines—mean S/N value and its confidence interval.

The highest value of tensile strength during a static tensile test was obtained when using the following independent variable parameters: (1) 3—0.24 mm, (2) 2—30°, (3) 4—100%, (4) 3—275 °C, (5) 1—concentric. The UTS value of test 10 was 38.46 MPa. The lowest value of tensile strength Rm during a static tensile test was obtained when using the following independent variable parameters: (1) 3—0.24 mm, (2) 3—60°, (3) 1—40%, (4) 2—270 °C, (5) 4—Archimedes spiral. The UTS value of sample 11 was 19.79 MPa. The highest value of the S/N ratio is achieved by sample 10—31.70, while the lowest by sample 11—25.93. The results of the Taguchi test analysis for individual printing parameters are presented graphically in Figure 7.

The result of the optimization is the highest value of the S/N ratio. During the established optimization criterion, which is the highest value of ultimate tensile strength, the printing process using the Original Prusa iMK3S+ 3D printer with Fiberlogy ABS material should use the following parameters: (1) layer height—0.22 mm; (2) orientation—90°; (3) filling ratio—100%; (4) nozzle temperature—275 °C; (5) type of surface layer finishing—octagonal spiral.

### 3.2. Acoustic Emission

**Figure 8:**
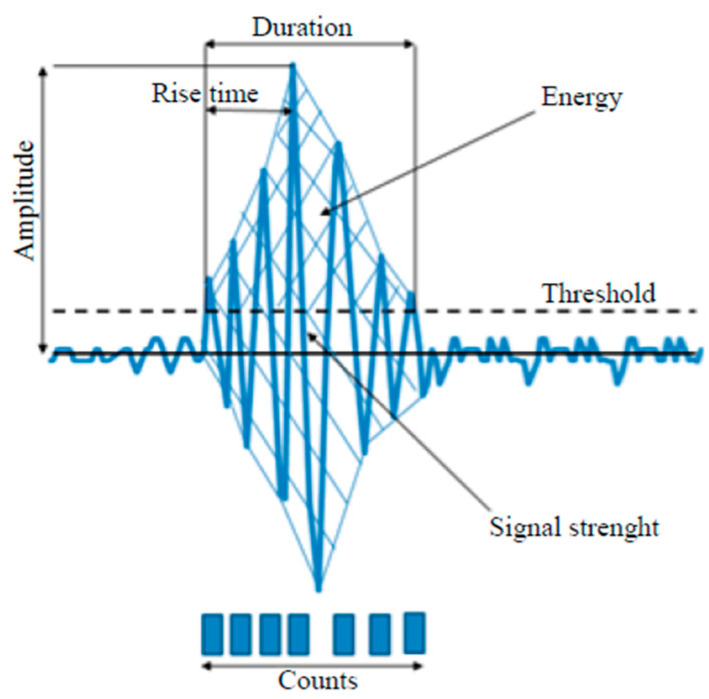
An example of an AE signal with characteristic parameters [13].

During AE studies, many parameters were recorded and analyzed. The most significant changes in selected parameters appeared above 15 s of the test duration. The following parameters were selected for analysis: amplitude, RMS, number of events, duration, rise time, signal energy, frequency. An example of an AE signal, along with the parameters characterizing it, is shown in Figure 8. Among the mentioned parameters describing the AE signal, the amplitude was selected for detailed analysis, as the parameter with the highest diagnostic value.

The analysis of AE signals recorded during the static tensile test was performed using AE Win for USB Version E5.30 software. Before starting the tests, the background acoustic noise from the testing machine was recorded to avoid analyzing signals not originating from the tested samples. In the article, graphical interpretations of the results are presented for samples selected in statistical tests, respectively: 10—the best, and 11—the worst.

**Figure 9:**
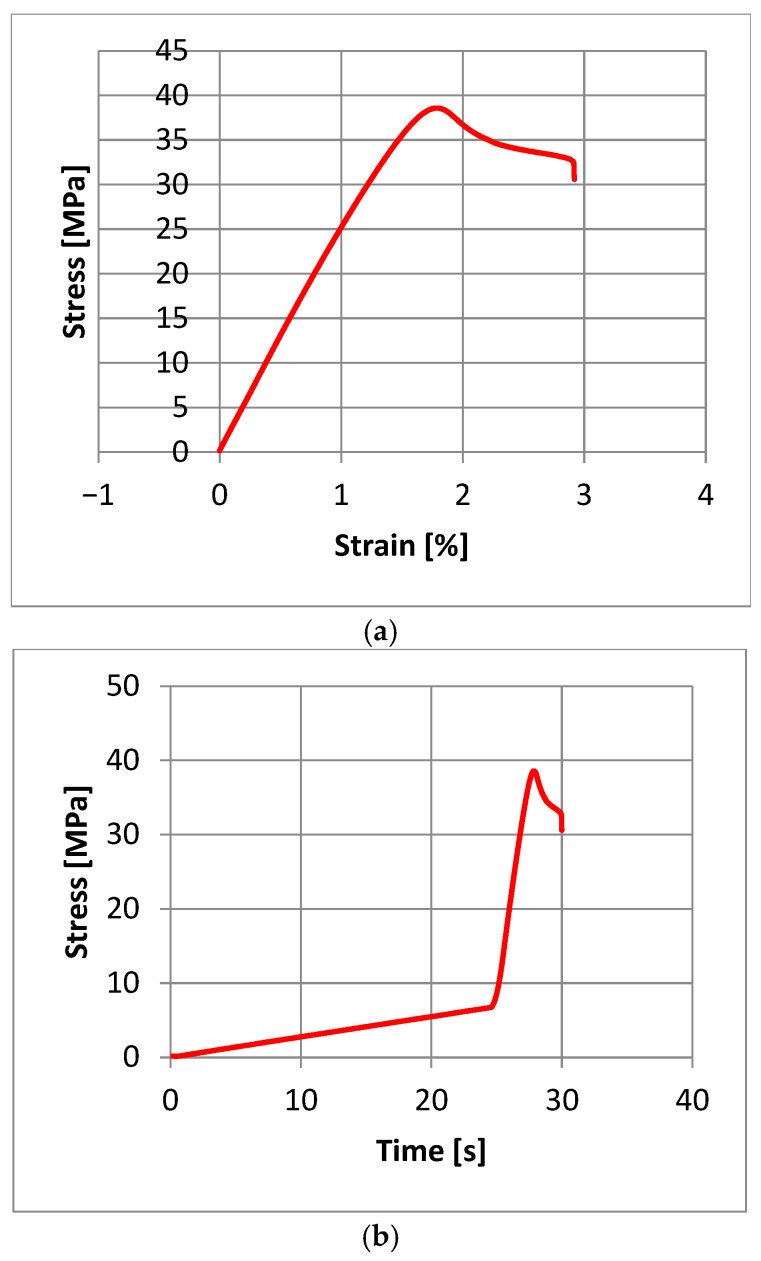
Graphs obtained from the static tensile test for sample no. 10: (**a**)—stress–strain, (**b**)—stress–time.

In a typical stress–strain diagram obtained in a static tensile test, we are not able to observe a clear yield point (Figure 9a). Changing the stress–strain diagram to a stress–time diagram (Figure 9b) allows us to observe the inflection point of the curve, which can be interpreted as a transition from an elastic to a visco-plastic state.

**Figure 10:**
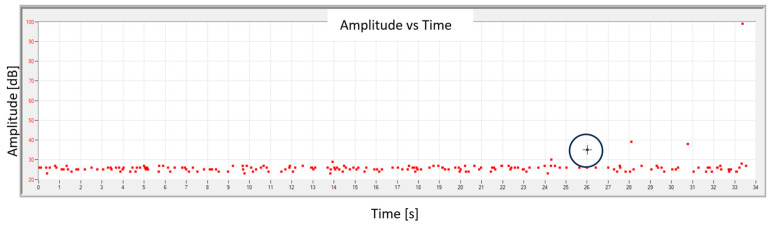
AE amplitude vs. time graph of sample No. 10.

Acoustic emission signals were recorded during the static tensile test. They were analyzed in software (AE Win with USB Version E5.30) dedicated to the equipment, and the graph of the signal amplitude change recorded as a function of time is presented in Figure 10. Based on previous research, it was established that achieving an AE signal with an amplitude exceeding 30 dB indicates the beginning of the material destruction process. The first point where the amplitude exceeds 30 dB is marked with a circle on the graph.

**Figure 11:**
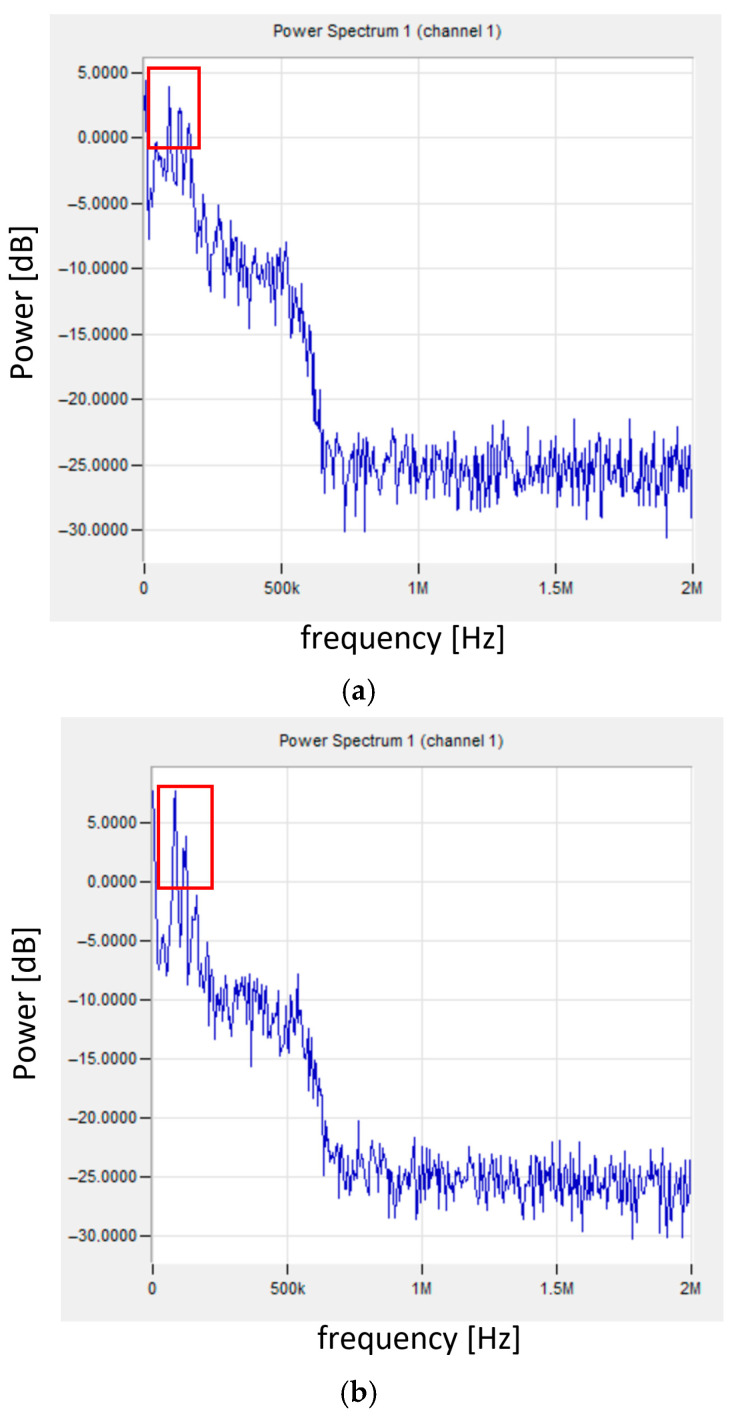

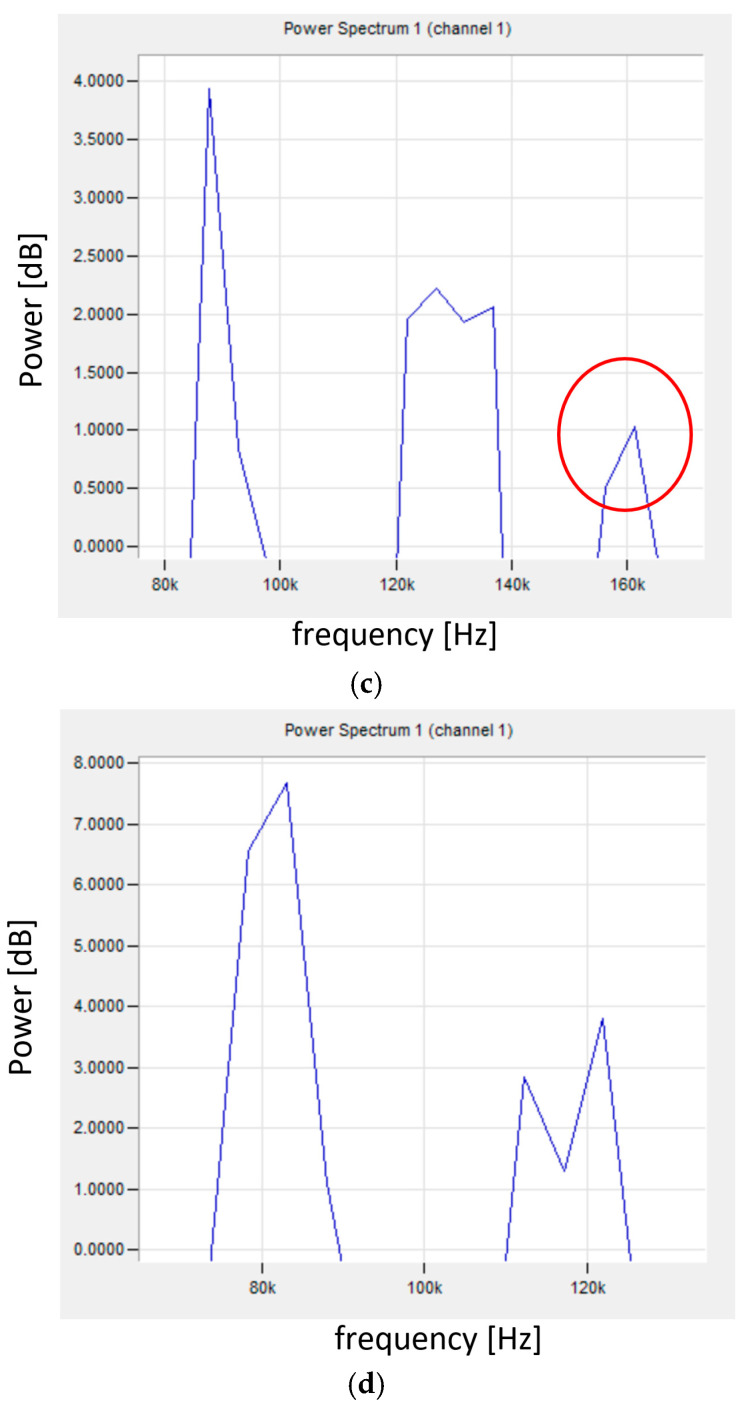
Frequency spectrum of the signal for sample no. 10: (**a**) for the selected transition point from the elastic to visco-plastic state (indicated in Figure 10); (**b**) for the signal in the range of elastic deformation; (**c**) enlargement of the selected area (**a**); (**d**) enlargement of the selected area (**b**).

Additionally, FFT analysis was performed for the selected signal. Figure 11 shows the frequency spectrum for the point indicated in Figure 10 and, for comparison, the spectrum for the signal recorded in the range of elastic deformation (before 26 s of the test).

Until the transition to the visco-elastic state, the signal had the character shown in Figure 11b,d, where the frequency changes in the range of 80–120 kHz can be observed. The relatively low signal frequencies indicate slowly changing processes accompanying elastic deformation. For the transition point from the elastic to the visco-plastic state, the signal frequency is in the range of 90–160 kHz (additional signal with 160 kHz—indicated by a circle in Figure 11c). The appearance of a higher frequency signal may indicate a sudden deformation of the material.

**Figure 12:**
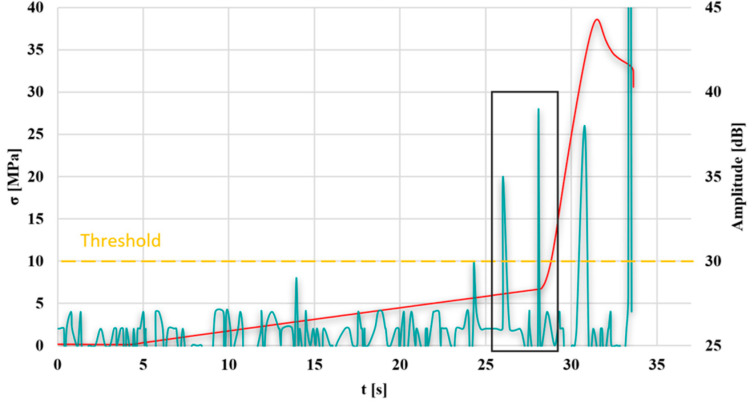
Graph of stress and AE amplitude as a function of time for the selected sample no. 10; green line—AE signal, red line—stress-strain diagram, black rectangle—time period between the transition from elastic to the visco-plastic detection by two methods.

To determine the stress value characteristic of the transition from the elastic state to the visco-plastic state, a stress–time diagram and an AE-amplitude-time diagram were plotted (Figure 12).

It was observed that the transition from the elastic to the visco-plastic state, determined based on AE, occurs approximately 2 s earlier compared to the stress–strain diagram. This amounts to 6.13 MPa for AE and 6.64 MPa for the static tensile test, respectively.

**Figure 13:**
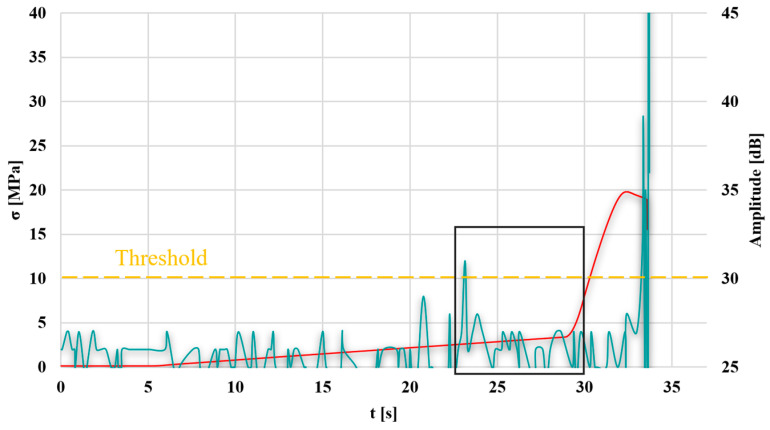
Graph of stress and AE amplitude as a function of time for the selected sample no. 11; green line—AE signal, red line—stress-strain diagram, black rectangle—time period between the transition from elastic to the visco-plastic detection by two methods.

Additionally, a graph is presented for sample 11, selected in the Taguchi method, as the worst selection of parameters (Figure 13), to compare the test results.

In the graph, the transition from the elastic to the visco-plastic state, determined based on AE, occurs approximately 5 s earlier compared to the stress–strain graph. This amounts to 2.83 MPa for AE and 4.84 MPa for the static tensile test, respectively.

The previously described signal overlay procedure was used for all trials. Table 5 shows the average values of the stress results for AE and the static tensile test. The highest and lowest values are marked in green and red, respectively.

The smallest difference in determining the transition from the elastic to visco-plastic state was obtained for test 10 and amounted to 0.51 MPa. This represents approximately 8% lower ultimate stress determined using the AE method compared to the analysis of data from a static tensile test (STT). The largest difference was determined for sample 11, which amounted to 2.01 MPa. In this case, the ultimate stress was lower by almost 50% when comparing the AE method to the STT method.

## 4. Conclusions

Analysis of variance shows that all independent variables have a statistically significant effect on tensile strength.

The sum of squares analysis shows that the degree of filling has the greatest influence on tensile strength, while the nozzle temperature has the least influence.

The highest value of tensile strength during a static tensile test was obtained for the following independent variable parameters: (1—layer height) 3—0.24 mm; (2—orientation) 2—30°; (3—filling ratio) 4—100%; (4—nozzle temperature) 3—275 °C; (5—type of filling of the surface layer); and 6—concentric.

The lowest value of tensile strength during a static tensile test was obtained for the following parameters of independent variables: (1—layer height) 3—0.24 mm; (2—orientation) 3—60°; (3—filling ratio) 1—40%; (4—nozzle temperature) 2—270 °C; (5—type of filling of the surface layer); and 6—Archimedes’ spiral.

The highest value of the S/N ratio was achieved by sample no. 10, and the lowest by sample no. 11.

Determining the strength properties of ABS thermoplastic based on changes in printing parameters, such as layer height, orientation, degree of filling, nozzle temperature, and type of surface layer, is crucial when used in the production of machine and device components. To obtain the highest values of tensile strength, elements should be printed using the Original Prusa iMK3S+ 3D printer with ABS material in the following settings of individual parameters: layer height—0.22 mm; orientation—90°; filling ratio—100%; nozzle temperature—275 °C; and type of surface layer—octagonal spiral.

To determine the permissible stresses during the operation of elements printed according to the proposed technology made of ABS material, the transition stress from the elastic to the visco-plastic state should be determined. A static tensile test, combined with the acoustic emission method, can be used to determine this stress.

Among the many recorded parameters describing the AE signal, the amplitude was selected for detailed analysis as the parameter with the highest diagnostic value. The limit value of the amplitude for determining significant changes in the tested materials was 30 dB.

Plotting the change in the amplitude of the acoustic emission signal as a function of time on a stress–time graph obtained from a static tensile test allowed for the determination of the stress value characteristic of the transition from the elastic to the visco-plastic state of the tested samples.

The first symptoms of the transition from the elastic to the visco-plastic state could be determined 2–5 s earlier in the case of the AE method compared to STT. This represents approximately 8–50% lower ultimate stress determined using the AE method compared to the analysis of data from a static tensile test for the best and worst test results.

The smallest difference in determining the transition from the elastic to the visco-plastic state was obtained for test 10 and amounted to 0.51 MPa, while the largest was obtained for test 11 and amounted to 2.01 MPa. This represents approximately 8% and 50% lower ultimate stress determined using the AE method compared to the analysis of data from a static tensile test.

For elements manufactured using the additive method from ABS material according to the proposed technology, the limiting stress value corresponding to the transition from the elastic to the visco-plastic state is approximately 6 MPa. To ensure the safe and reliable operation of parts manufactured using the MEX method from ABS material, the load range should not exceed this value.

The novelty of this study is the use of acoustic emission monitoring simultaneously with static tensile testing of material extrusion (MEX)—fabricated ABS polymer components. A literature review revealed a clear research gap in this area, which motivated the authors to undertake this study. A comparison with results obtained for ABS samples manufactured using conventional injection molding is planned. Additionally, other additively manufactured materials will be investigated for their potential use in various environments, including marine environments.

## Figures and Tables

**Table 1:** Printing process parameters.

Parameters	Level 1	Level 2	Level 3	Level 4
Layer height [mm]	0.20	0.22	0.24	0.26
Orientation [°]	0	30	60	90
Filling ratio [%]	40	60	80	100
Nozzle temp. [°C]	265	270	275	280
Type of finishing of the top layer	Concentirc	Parallel line	Octagonal spiral	Archimedes spiral

**Table 2:** Orthogonal array showing the design of Taguchi L16 experiment along with the variables.

Trial Number	Layer Height [mm]	Orientation [°]	Filling Ratio [%]	Nozzle Temp. [°C]	Type of Finishing of the Top Layer
1.	0.20	0	40	265	Concentric
2.	0.20	30	60	270	Parallel line
3.	0.20	60	80	275	Octagonal spiral
4.	0.20	90	100	280	Archimedes spiral
5.	0.22	0	60	275	Archimedes spiral
6.	0.22	30	40	275	Octagonal spiral
7.	0.22	60	100	265	Parallel line
8.	0.22	90	80	270	Concetric
9.	0.24	0	80	280	Parallel line
10.	0.24	30	100	275	Concentric
11.	0.24	60	40	270	Archimedes spiral
12.	0.24	90	60	265	Octagonal spiral
13.	0.26	0	100	270	Octagonal spiral
14.	0.26	30	80	265	Archimedes spiral
15.	0.26	60	60	280	Concetric
16.	0.26	90	40	275	Parallel line

**Table 3:** Variance analysis of input parameters.

Independent Variable	SS Sum of Squares	F Fisher Test	*p* Significance Factor
Layer height [mm]	0.8365	60.78	<0.01
Orientation [°]	2.3584	171.35	<0.01
Filling ratio [%]	186.1050	13,521.84	<0.01
Nozzle temp. [°C]	0.4509	32.76	<0.01
Type of finishing of the top layer	0.7602	55.24	<0.01
Rest	0.1468		

**Table 4:** Averaged tensile strength results and S/N ratio (highlighted in green—best, red—worst).

Trial Number	Layer Height [mm]	Orientation [°]	Filling Ratio [%]	Nozzle Temp. [°C]	Type of Finishing of the Top Layer [-]	UTS [MPa]	S/N
Independent Variable	(1)	(2)	(3)	(4)	(5)		
1	1	1	1	1	1	21.46	25.96
2	1	2	2	2	2	22.15	26.90
3	1	3	3	3	3	25.26	28.04
4	1	4	4	4	4	37.01	31.36
5	2	1	2	3	4	23.67	27.48
6	2	2	1	4	3	20.25	26.12
7	2	3	4	1	2	38.04	31.60
8	2	4	3	2	1	27.81	28.89
9	3	1	3	4	2	27.27	28.71
10	3	2	4	3	1	38.46	31.70
11	3	3	1	2	4	19.79	25.93
12	3	4	2	1	3	23.71	27.50
13	4	1	4	2	3	37.22	31.41
14	4	2	3	1	4	25.27	28.05
15	4	3	2	4	1	21.86	26.80
16	4	4	1	3	2	21.55	26.65

**Table 5:** Average values of AE stresses and static tensile tests (highlighted in green—best, red—worst).

Trial Number	Stress Determined in AE [MPa]	Stress Determined by Static Tensile Test [MPa]
1	2.69	4.39
2	2.73	4.24
3	3.21	4.33
4	5.19	6.35
5	2.85	4.06
6	2.64	4.37
7	5.44	6.53
8	3.98	5.40
9	3.39	4.68
10	6.13	6.64
11	2.83	4.84
12	2.83	4.22
13	5.26	6.39
14	2.76	4.25
15	2.71	4.20
16	2.74	4.32

## Data Availability

The original contributions presented in this study are included in the article. Further inquiries can be directed to the corresponding author.
